# Supplementary material for: Quanty-cFOS, a Novel ImageJ/Fiji Algorithm for Automated Counting of Immunoreactive Cells in Tissue Sections
Source: Cells. 2023 Feb 23;12(5):704. doi: 10.3390/cells12050704 (PMC10000431; doi:10.3390/cells12050704)
Supplement: Supplementary file 1 [file cells-12-00704-s001.zip › Beretta et al Supplementary information.pdf]

Beretta et al., Supplementary information:

Supplementary figures and legends:

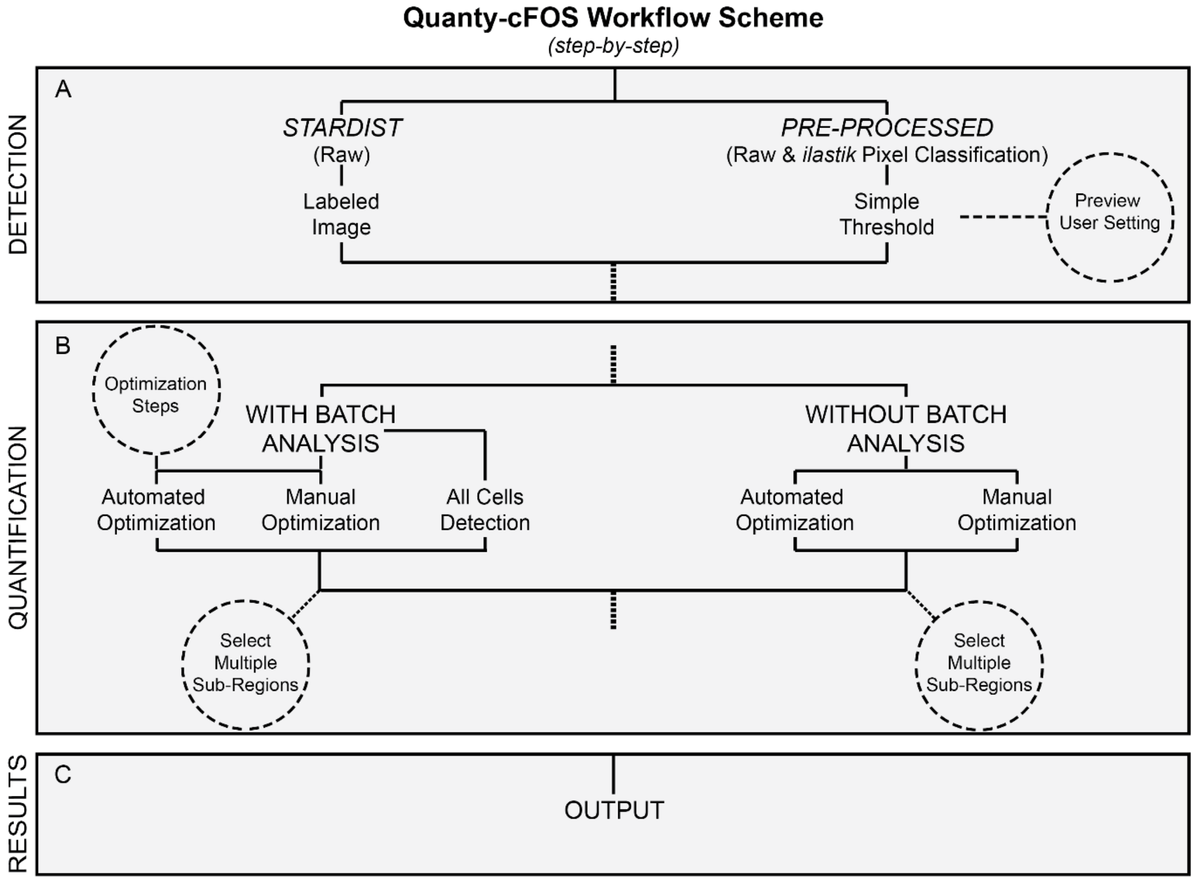

Figure S1. Step-by step workflow description and applications of Quanty-cFOS.
(A) Quanty-cFOS-supported detection methods for Fos protein and *c-fos* mRNA counting. StarDist cell segmentation is performed with one input directory containing the raw images shown for Fos protein cell counting. Pre-processed cell detection with two input directories, the raw images input directory and the pre-processed images input directory shown for *c-fos* mRNA *in-situ* cell counting (e.g., ilastik pixel classification Probability Map images). *Preview User Setting* can be used to manually find the best threshold method in ImageJ/Fiji to threshold the pre-processed image. (B) Quantification with and without batch analysis. *With Batch Analysis* option supports: *Automated Optimization, Manual Optimization* and *All Cells Detection*. '*Optimization steps'* are the number of images used to compute the intensity threshold cutoff in the order of opening. This parameter is omitted when the *All Cells Counts* method is used. *Without Batch Analysis* option supports: *Automated Optimization* and *Manual Optimization options,* such that each image is processed independently. The user can switch between the automated and the manual method any time a new image is processed. *Select Multiple ROIs* represents a function whereby user can select specific subregions in the input image and quantify specific sub-regions within an image, if necessary. (C) Results are saved in the output root directory created by the Quanty-cFOS tool.

Quanty-cFOS Automated and Manual Optimization Scheme

(step-by-step)

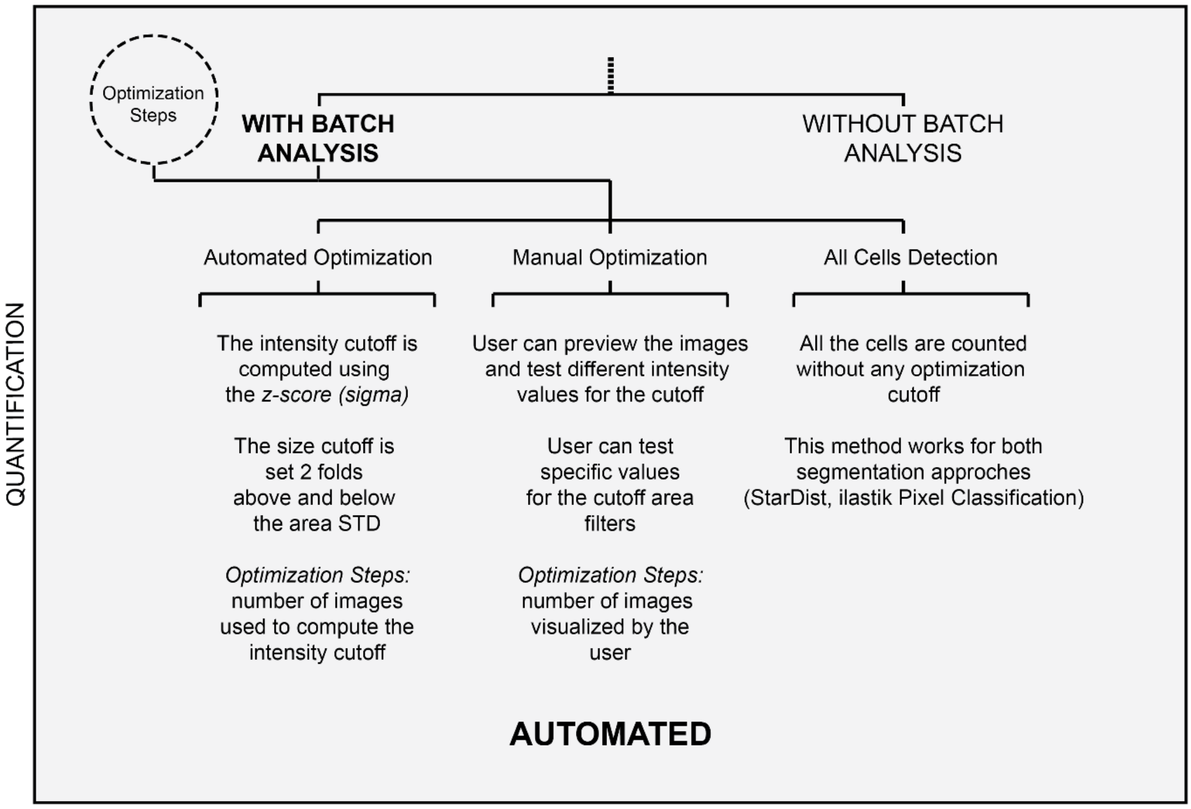

Figure S2. Automated Quanty-cFOS with batch analysis.
Batch Analysis With Automated Optimization: the mean intensity value is used to compute the intensity threshold cutoff using the number of images specified by the *Optimization steps* in the opening order. The area cutoff is used to exclude cells above and below 2 times the standard deviation (S.D.) of that area. *Batch Analysis With Manual Optimization*: the user can visualize the number of images specified by the *Optimization Steps* and change the intensity threshold of each image to refine the intensity cutoff**.** Cell area can be measured on the displayed images and used as a size filter to exclude cells above and below the area cutoff value.
Batch Analysis With All Cells Detection is used to count all the cells in the images without intensity cutoff. The *Optimization Steps* parameter is not used. Both detection methods for image segmentation are supported.

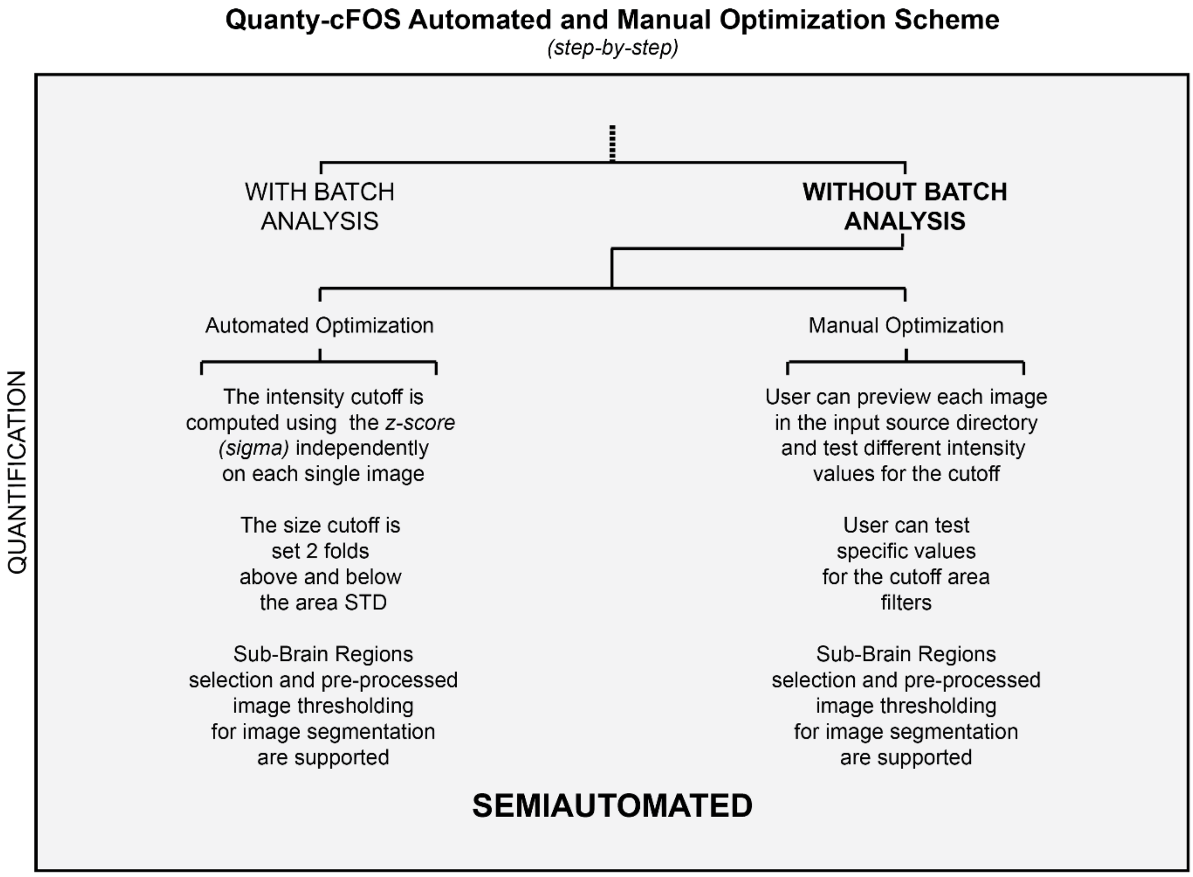

Figure S3. Semi-automated Quanty-cFOS without batch analysis
Automated Optimization Without Batch Analysis: this functionality can be used to count Fos/*c-fos*-positive cells on single images. The z-score value is performed independently on each image and used to compute the intensity cutoff. The area cutoff is used to exclude cells below and above the area value. This option allows to use the *Sub-Brain Regions* selection tool to count positive cells in different brain areas of the same input image. To estimate the intensity cutoff all the segmented cells in the image are used.
Manual Optimization Without Batch Analysis: the user can visualize each image and adjust manually the intensity cutoff and the area cutoff. The *Sub-Brain Regions* options can be used to process specific subregions of interest in the input image. Both detection methods for image segmentation are supported.

Supplementary video's legends:

Movie S1. Quanty-cFOS all cells counting with batch processing without intensity cutoff.
Quanty-cFOS demo video for counting all cells without intensity threshold cutoff. The video shows how to use the Quanty-cFOS.ijm ImageJ/Fiji tool to detect all the cells in the input raw images. Cell segmentation is performed using the *StarDist 2D Versatile Fluorescence Nuclei* model for demonstration purposes. *Ilastik pixel classification* or any pre-processing step can be used to achieve the same result. The *Optimization Steps* are omitted, and the *Batch Analysis* box needs to be checked. The user needs to select the input raw directory with the raw images to process. All cells counting can be performed by unchecking the *Automated* and *Manual Optimization* box in the *User Input Setting Window*. The *Sigma* value is not considered for all cells counting analysis. Results are saved in the output directory created outside the selected input path (MacroResults_YYMMDD_SS; YY: year, MM: month, DD: day and SS: second). Each processed image is saved in an output subdirectory created by Quanty-cFOS and located in the main output path named with the input raw image file name. The ImageJ/Fiji ROIs of each detected cell, the segmented image (labeled image, color coded – Glasbey Inverted ImageJ/Fiji Lookup Table) and the center of mass are saved as csv file. The Quanty-cFOS processing steps are saved in the *Log.txt* file and a *SummaryMeasurements.csv* file is created to summarize the number of cells in each processed image. For all cells counting, positive and negative columns are set on NaN. An additional *LabeledImages* folder is generated in the main output path and contains all the images with the labeled segmented cells to simplify further analysis.

Movie S2. Quanty-cFOS automated intensity cutoff workflow using StarDist segmentation method for Fos protein cell counting.
Quanty-cFOS demo video with Automated Optimization for Fos protein counting. The video shows how to use the Quanty-cFOS.ijm ImageJ/Fiji tool to detect Fos-positive neurons with a mean intensity value above the computed threshold cutoff. Cell segmentation is performed using the *StarDist 2D Versatile Fluorescence Nuclei* model for demonstration purposes. *Ilastik pixel classification* or any pre-processing step can be used to achieve the same result. The *Optimization Steps* are set on 3 for demo purpose, and the *Batch Analysis* box is checked. We recommend to use at least 30% of the input images to get a proper estimation of the intensity cutoff.
The user needs to select the input raw directory with the raw images to process. Automated counting can be achieved by checking the *Automated Optimization* box in the *User Input Setting Window*. The Default *Sigma* value used to compute the z-score is set to 3. This value can be changed to achieve a better counting. The computed cutoff intensity value is plotted at the end of the processing. Results are saved in the output directory created outside the selected input path (MacroResults_YYMMDD_SS; YY: year, MM: month, DD: day and SS: second). Each processed image is saved in an output subdirectory created by Quanty-cFOS and located in the main output path named with the input raw image file name. The ImageJ/Fiji ROIs of each detected cell, the segmented positive and negative cells (labeled image; 2 colored output) and the center of mass are saved as csv file. ROIs for Fos-positive neurons are highlighted in green and red ROIs show the Fos-negative neurons. The Quanty-cFOS processing steps are saved in the *Log.txt* file and a *SummaryMeasurements.csv* file is created to summarize the number of cells counted in each processed image. An additional *LabeledImages* folder is generated in the main output path and contains all the images with the labeled positive and negative neurons to simplify further analysis.

Movie S3. Quanty-cFOS manual intensity cutoff workflow using StarDist segmentation method for Fos protein cell counting.
Quanty-cFOS demo video with cFOS Manual Optimization for Fos protein counting. The video shows how to use the Quanty-cFOS.ijm ImageJ/Fiji tool to detect Fos-positive neurons with a mean intensity value above the manual threshold cutoff. Cell segmentation is performed using the *StarDist 2D Versatile Fluorescence Nuclei* model for demonstration purposes. *Ilastik pixel classification* or any pre-processing step can be used to achieve the same result. The *Optimization Steps* are set on 3 for demo purpose, and

the *Batch Analysis* box is checked. We recommend to use at least 10% of the input images to get a proper estimation of the intensity cutoff.
The user needs to select the input raw directory with the raw images to process. Counting with a manual intensity cutoff can be achieved by checking the *Manual Optimization* box in the *User Input Setting Window*. The Default *Sigma* value used to compute the z-score is set to 3. If necessary, this value can be changed to achieve a better counting. Depending on the number of *Optimization Steps*, the user can specify the best threshold to compute the Fos intensity cutoff on the previewed images. Cell area can also be used to exclude cells below and above the cell size. Results are saved in the output directory created outside the selected input path (MacroResults_YYMMDD_SS; YY: year, MM: month, DD: day and SS: second). Each processed image is saved in an output subdirectory created by the Quanty-cFOS and located in the main output path named with the input raw image file name. Indeed, the ImageJ/Fiji ROIs of each detected cell, the segmented positive and negative cells (labeled image; 2 colored output) and the center of mass are saved as csv file. ROIs for Fos-positive neurons are highlighted in green and red ROIs show the Fos-negative neurons. The Quanty-cFOS processing steps are saved in the *Log.txt* file and a *SummaryMeasurements.csv* file is created to summarize the number of cells counted in each processed image. An additional *LabeledImages* folder is generated in the main output path and contains all the images with the labeled positive and negative neurons to simplify further analysis.

Movie S4. Quanty-cFOS automated intensity cutoff workflow using raw images and ilastik pixel classification probability map images for *c-fos* mRNA cell counting.
Quanty-cFOS demo video with Automated Optimization for counting cells positive for *c-fos* mRNA. The video shows how to use the Quanty-cFOS.ijm ImageJ/Fiji tool to detect *c-fos* -positive neurons with a mean intensity value above the computed threshold cutoff. Cell segmentation is performed on the *Ilastik pixel classification* probability map images and the intensity cutoff value is computed on the raw images. The *Optimization Steps* are set on 3 for demo purpose, and the *Batch Analysis* box is checked. We recommend to use at least 30% to 40% of the input images to get a proper estimation of the intensity cutoff.
The user needs to select the input raw directory with the raw images and the ilastik pixel classification probability map directory containing the corresponding images to process. Automated counting can be achieved by checking the *Automated Optimization* box in the *User Input Setting Window*. The Default *Sigma* value used to compute the z-score is set to 3. If necessary, this value can be changed to achieve a better counting. The computed cutoff intensity value is plotted at the end of the processing. Results are saved in the output directory created outside the selected input path (MacroResults_YYMMDD_SS; YY: year, MM: month, DD: day and SS: second). Each processed image is saved in an output subdirectory created by Quanty-cFOS and located in the main output path named with the input raw image file name. The ImageJ/Fiji ROIs of each detected cell, the segmented positive and negative cells (labeled image; 2 colored output) and the center of mass are saved as csv file. ROIs for positive *c-fos*-positive neurons are highlighted in green and red ROIs show the *c-fos*-negative neurons. Quanty-cFOS processing steps are saved in the *Log.txt* file and a *SummaryMeasurements.csv* file is created to summarize the number of cells counted in each processed image. An additional *LabeledImages* folder is generated in the main output path and contains all the images with the labeled positive and negative neurons to simplify further analysis.

Movie S5. Quanty-cFOS without batch analysis with multiple brain sub-regions selection
Quanty-cFOS demo video for Fos protein cell counting without batch analysis with multiple selected ROIs to count cells in specific regions of an image. The workflow can be used only *Without Batch Analysis,* and the *Optimization Steps* are omitted. The user needs to select an input directory with the raw images to process. Multiple sub-brain regions cell counting can be achieved by checking the *Select Multiple Sub-Brain Regions* box in the *User Input Setting Window*. For this demo video, we chose *Automated Optimization*; however, the optimized intensity cutoff is computed independently for each image. The user can draw one or more ROIs, with any shape, and the cell counting is computed only on the selected sub-brain regions. For instance, Fos-positive cell counting is performed on the first ROI,

the user can select a second ROI to count cells in a different brain region of the same image. After an image is processed, the *User Input Setting Window* pops up again and the user can decide to process multiple sub-regions in the next image or count all the cells with the selected method (e.g., Automated Optimization). The iterative process is repeated for all the images in the input source directory. Results are saved in the output directory created outside the selected input path (MacroResults_YYMMDD_SS; YY: year, MM: month, DD: day and SS: second). Each processed image is saved in an output subdirectory created by the Quanty-cFOS and located in the main output path named with the input raw image file name. Indeed, the ImageJ/Fiji ROIs of each detected cell, the segmented positive and negative cells (labeled image; 2 colored output) and the center of mass are saved as csv file for the specific sub-brain regions or for all the cells in the image. ROIs for positive Fos neurons are highlighted in green and red ROIs show the Fos negative neurons. Quanty-cFOS processing steps are saved in the *Log.txt* file and a *SummaryMeasurements.csv* file is created to summarize the number of cells counted in each subregion and/or in each processed image. An additional *LabeledImages* folder is generated in the main output path and contains all the images with the labeled positive and negative neurons to simplify further analysis. This workflow can be adapted to count *c-fos* mRNA-positive cells.
